# Brain Connectomics' Modification to Clarify Motor and Nonmotor Features of Myotonic Dystrophy Type 1

**DOI:** 10.1155/2016/2696085

**Published:** 2016-05-25

**Authors:** Laura Serra, Matteo Mancini, Gabriella Silvestri, Antonio Petrucci, Marcella Masciullo, Barbara Spanò, Mario Torso, Chiara Mastropasqua, Manlio Giacanelli, Carlo Caltagirone, Mara Cercignani, Giovanni Meola, Marco Bozzali

**Affiliations:** ^1^Neuroimaging Laboratory, IRCCS Santa Lucia Foundation, Via Ardeatina 306, 00179 Rome, Italy; ^2^Department of Engineering, “Roma Tre” University, Via Vito Volterra 62, 00154 Rome, Italy; ^3^Department of Geriatrics, Orthopedics and Neuroscience, Institute of Neurology, Catholic University of Sacred Heart, Largo Agostino Gemelli 8, 00168 Rome, Italy; ^4^UOC Neurologia e Neurofisiopatologia, AO San Camillo Forlanini, Via Portuense 332, 00149 Rome, Italy; ^5^SPInal REhabilitation Lab (SPIRE), IRCCS Santa Lucia Foundation, Via Ardeatina 306, 00179 Rome, Italy; ^6^Department of Clinical and Behavioural Neurology, IRCCS Santa Lucia Foundation, Via Ardeatina 306, 00179 Rome, Italy; ^7^Department of Neuroscience, University of Rome “Tor Vergata”, Via Montpellier No. 1, 00133 Rome, Italy; ^8^Clinical Imaging Sciences Centre, Brighton & Sussex Medical School, University of Sussex, Falmer, Brighton, East Sussex BN1 9RR, UK; ^9^Department of Neurology, IRCCS Policlinico San Donato, University of Milan, Via Morandi 30, San Donato Milanese, 20097 Milan, Italy

## Abstract

The adult form of myotonic dystrophy type 1 (DM1) presents with paradoxical inconsistencies between severity of brain damage, relative preservation of cognition, and failure in everyday life. This study, based on the assessment of brain connectivity and mechanisms of plasticity, aimed at reconciling these conflicting issues. Resting-state functional MRI and graph theoretical methods of analysis were used to assess brain topological features in a large cohort of patients with DM1. Patients, compared to controls, revealed reduced connectivity in a large frontoparietal network that correlated with their isolated impairment in visuospatial reasoning. Despite a global preservation of the topological properties, peculiar patterns of frontal disconnection and increased parietal-cerebellar connectivity were also identified in patients' brains. The balance between loss of connectivity and compensatory mechanisms in different brain networks might explain the paradoxical mismatch between structural brain damage and minimal cognitive deficits observed in these patients. This study provides a comprehensive assessment of brain abnormalities that fit well with both motor and nonmotor clinical features experienced by patients in their everyday life. The current findings suggest that measures of functional connectivity may offer the possibility of characterizing individual patients with the potential to become a clinical tool.

## 1. Introduction

Myotonic dystrophy type 1 (DM1) is the most common muscular dystrophy observed in adults [1]. It is caused by a CTG triplet repeat expansion within the myotonic dystrophy protein kinase (DMPK) gene located on chromosome 19q13.3, whose inheritance is autosomal dominant [2]. DM1 is a multisystemic disorder dominated by muscular impairment but involving also other organs including the brain [1].

It is becoming increasingly clearer that most of the impairment observed in patients with DM1 is driven by higher-level dysfunctions [3–7]. DM1 brains have been demonstrated to be structurally damaged in both tissues, the grey (GM) and white matter (WM) [7–11], with a specific anatomical distribution of abnormalities. This structural damage has been consistently reported across independent studies [7–11], and it was more recently associated with CTG triplet expansions in the DMPK gene and measures of clinical severity [7]. Abnormal patterns of brain connectivity have also been reported in DM1 patients and have been demonstrated to account for patients' personality traits [6]. While mental retardation is frequently observed in the congenital form of DM1 [12] the adult forms are typically characterized by isolated cognitive deficits [7, 13]. This relative preservation of global cognition contrasts with the pathological and neuroimaging evidence of diffuse WM abnormalities [7–10] and with the paradoxical failure of these patients in everyday life. In addition, upon investigating functional connectivity within the so-called default mode network, whose disruption is typically associated with cognitive impairment in degenerative dementia [14, 15], DM1 patients reveal an increase of connectivity in some critical nodes [6]. These data, taken altogether, suggest DM1 as neurodevelopmental disorder that associates with peculiar rearrangements in neuronal networks' segregation and integration properties. These complex alterations, which are hardly detectable by structural brain assessments, are likely to account for the distinctive motor and nonmotor features observed in DM1. Resting-state functional MRI (RS-fMRI) [16] is one of the most widely used methods to investigate brain connectivity in neurological and psychiatric diseases [17, 18], with the advantage of not requiring participants to perform any active task. RS-fMRI data can be analysed using different methodological approaches. One promising technique is based on the whole brain analysis driven by graph theory [19], a mathematical approach that describes complex systems as networks [20]. In simple words, the brain is conceptualized as a number of regions (nodes) that are functionally connected to each other by edges and whose importance and efficiency within the whole network are determined by their functional specialization (i.e., segregation) and integration. In this view, some nodes are more critical (i.e., centrality) for information processing (efficiency in information transferring) and are called “hubs.” Abnormal connectivity between “hubs” is believed to cause more deficits than that between peripheral nodes [19, 20]. With these concepts in mind, the current work aimed at assessing topological properties of DM1 brains, their potential relationship with genetics, and their ability in accounting for patients' motor and nonmotor clinical features. To the best of our knowledge, this is the first attempt to investigate brain connectomics in DM1 patients.

## 2. Materials and Methods

### 2.1. Participants

Thirty-one patients with a molecular diagnosis of DM1 were recruited from the Neuromuscular and Neurological Rare Diseases Center at San Camillo Forlanini Hospital (Rome, Italy) and the Institute of Neurology at the Catholic University of Rome (Rome, Italy). Data from part of this patients' cohort were previously presented in an independent study [6]. Twenty-six healthy participants were recruited from Santa Lucia Foundation in Rome (Italy). As detailed below, CTG expansion size within the DMPK gene was assessed for DM1 participants and used to classify them according to the International Myotonic Dystrophy Consortium nomenclature [22]. Demographic characteristics of the participants are summarized in Table 1. Principal genetic and clinical characteristics of DM1 patients are summarized in Table 2. All participants were right-handed as assessed by the Edinburgh Handedness Inventory [23]. All subjects underwent clinical assessment to exclude the presence of major systemic and neurological illnesses in controls and pathologies different from known comorbidities in DM1 patients. The study was approved by the Ethical Committee of Santa Lucia Foundation and written informed consent was obtained from all participants before study initiation. All procedures performed in this study were in accordance with the 1964 Helsinki declaration and its later amendments or comparable ethical standards.

### 2.2. Genetic Assessment

Normal and protomutated alleles were analysed using “touchdown” PCR on DNA obtained from peripheral blood leukocytes (PBL). Briefly, 50 pg of PBL-DNA was amplified in a 20 *μ*L volume with fluorescent labelled primer 101 and primer 102. Reactions were cycled through eight rounds at 94°C (30′′), 68°C (30′′) (−1°C per cycle), and 72°C (30′′), followed by 30 rounds at 94°C (30′′), 60°C (30′′), and 72°C (30′′). PCR products were then analysed using an ABI PRISM 310 Genetic Analyzer. Determination of expanded alleles was performed on 10 pg of PBL-DNA, which underwent XL-PCR1 and 1% agarose gel electrophoresis. PCR products were analysed by Southern blotting with subsequent hybridization to a 32P radiolabeled (CTG) 7-oligonucleotide probe and detected using autoradiography.

### 2.3. Neuropsychological Assessment

All participants underwent an extensive neuropsychological battery covering all cognitive domains, which included (a) Mini Mental State Examination (MMSE) [24]; (b) Rey's 15-Word List (Immediate and 15-min Delayed recall) [25] to assess episodic verbal memory; (c) Digit Span and Corsi Block Tapping task [26] forward and backward as measures of short-term memory; (d) naming objects and verbs subtests of the BADA (“Batteria per l'Analisi dei Deficit Afasici,” Italian for “Battery for the analysis of aphasic deficits”) [27] to assess language abilities; (e) Raven's Coloured Progressive Matrices [25] to assess reasoning; (f) copy of simple drawings with and without landmarks [25] to evaluate constructional abilities; and (g) Phonological Word Fluency [25] and Stroop test [28] to explore executive functions. Italian normative data were used for all tests both for score adjustment (gender, age, and education) and for defining normality cut-off scores (95% tolerance interval). A series of thirteen one-way ANOVAs were used to test differences in cognitive performance between patients and controls (*p* values < 0.004 after Bonferroni's correction). Additionally, in the patient group, MMSE scores were correlated with CTG triplet expansion, age of clinical onset, and Muscular Impairment Rating Scale (MIRS) [29] scores (*p* values < 0.02 after Bonferroni's correction). Statistical analyses were performed using SPSS-20 (SPSS Inc., Chicago, Illinois).

### 2.4. Image Acquisition and Preprocessing of Resting-State fMRI

All participants underwent MRI at 3 T, including (1) 3D Modified Driven Equilibrium Fourier Transform (MDEFT) scan (TR = 1338 ms, TE = 2.4 ms, matrix = 256 × 224, *n*. slices = 176, and thickness = 1 mm); (2) T2^*∗*^ weighted echo planar imaging (EPI) sensitized to blood oxygenation level dependent (BOLD) contrast (TR = 2080 ms, TE = 30 ms, 32 axial slices parallel to AC-PC line, matrix = 64 × 64, pixel size = 3 × 3 mm^2^, slice thickness = 2.5 mm, and flip angle: 70°) for resting-state fMRI (RS-fMRI). BOLD EPI images were collected during rest for 7 min and 20 s, resulting in a total of 220 volumes. During this acquisition, participants were instructed to keep their eyes closed, not to think of anything in particular, and not to fall asleep. EPI images were preprocessed for resting-state fMRI using Statistical Parametric Mapping 8 (SPM8 http://www.fil.ion.ucl.ac.uk/spm/) and in-house MATLAB scripts.

The first 4 volumes of each fMRI time series were discarded to allow for T1 equilibration effects; then, images underwent head motion correction (using the standard SPM8 realignment algorithm), compensation for slice-dependent time shifts, and coregistration to the corresponding MDEFT. Each MDEFT-volume was segmented using the standard SPM8 algorithm and the resulting grey matter images were used to compute each participant's total grey matter volume. Segmentation derived normalization parameters were used to warp the motion and slice-time corrected EPI images into Montreal Neurological Institute (MNI) coordinates. In-house software was used to remove the global temporal drift using a 3rd-order polynomial fit. Data were then filtered by regressing out movement vectors, average white matter, and cerebrospinal fluid signal. EPI images were then filtered using a phase-insensitive band-pass filter (passband 0.01–0.08 Hz) to reduce effects of low frequency drift and high frequency physiological noise and then smoothed with an 8 mm^3^ FWHM 3D Gaussian Kernel.

### 2.5. Construction of Connectivity Matrices

In order to define brain nodes, for each participant, the whole brain was parcellated into 116 regions of interests (ROIs) applying an automated anatomical labelling (AAL) atlas adapted to the EPI space using linear registration. Each ROI corresponds to a node of the network, and its mean time course was calculated as the average of the fMRI time series from all voxels within the region. Correlation matrices (i.e., adjacency matrices) were then obtained calculating the correlation between all pairs of ROI mean signals. The resulting adjacency matrices were analysed using 2 complementary approaches: (1) network-based statistics [30], which compares between groups the strength of connectivity for each pair of nodes in the network, thus assessing the presence of subnetwork altered connectivity in patients and (2) graph theory, which characterizes the network using special indices describing its shape and properties, including efficiency and resilience to attack (Brain Connectivity Toolbox) [19].

### 2.6. Networks-Based Analysis

This analysis was preformed using the “networks-based statistics” (NBS) tool developed by Zalesky and coauthors [30] and allowed testing for any significant difference of inter-Nodal functional connectivity (in all possible pairwise associations) between DM1 patients and controls. Between-group comparison was based on a two-sample *t*-test, using 50000 permutations and setting the significant *p* value at 0.005 (NBS-connectome corrected for multiple comparisons). Nodes with higher number of connections were considered more crucial for information processing and defined as “hubs.” According to previous literature [31], we considered “hubs” those nodes whose number of inter-Nodal connections exceeded one standard deviation the mean number of connections for every node within the significant network (estimated by NBS).

Then, using the NBS toolbox, for each DM1 patient, we extracted the “extent of connectivity” (a measure of connectivity strength within disrupted networks) to be used for correlations with clinical/genetic and neuropsychological variables. For the choice of the most appropriate statistical approach (parametric versus nonparametric correlation test), the normality of data distribution was assessed using the Shapiro-Wilk test (*W*). MIRS score and CTG triplet expansion were chosen as clinical/genetic variables in the correlation analyses with extent of connectivity (Bonferroni's correction: *α* = 0.05/2, *p* = 0.025). All neuropsychological scores were used as cognitive variables for the same correlation analyses (Bonferroni's correction: *α* = 0.05/13, *p* = 0.004).

### 2.7. Graph Theory Analysis

A connectivity matrix is a representation of a graph, where the nodes correspond to the rows and columns of the matrix and the edges are characterized by the values at the intersection of rows and columns. We used Brain Connectivity Toolbox [19] and MATLAB custom scripts to explore global and local topological properties of each participant's brain. Undirected binary connectivity matrices were built as thresholding adjacency matrices with different correlation values for each subject. The reason is that the choice of a single common threshold across different subjects leads to comparing networks with different densities, where density is defined as the ratio between the total number of edges and the maximum possible number of edges in the network. In order to prevent spurious differences in the network topologies due to different density values [32], multiple correlation thresholds were used for each subject, obtaining several connectivity matrices for each subject in a specific density range. In this way, subjects could be compared by means of connectivity matrices with the same density values. The density range was determined checking at each density value both the absence of isolated nodes and the presence of small-world properties (i.e., most nodes can be reached from any other by a small number of steps). In our case, the lower and the upper limits of this range are, respectively, 0.19 and 0.47. We chose to use a range step of 0.01 in line with other clinical studies [33, 34].

Details of graph theory and its application to brain networks can be found in previous papers [19, 20, 35] together with a full description of all the indices that can be derived from it. For the purposes of the current investigation, we focused on the topological measures with a more direct clinical interpretation [33–38].

As global metrics, mean Clustering Coefficient (i.e., the fraction of one node's neighbours that are also neighbours of each other), Characteristic Path Length (i.e., the average length of sequences of nodes that form routes), Assortativity (an index of the likelihood for high-degree nodes to be linked together), and Modularity (a measure of the presence of a community structure in a network) were calculated [19]. In order to further avoid spurious results, Normalized Clustering Coefficient and path length were also employed. The normalization was performed evaluating the ratio between the original metrics and those derived from random networks. Then, the ratio between the normalized mean Clustering Coefficient and the normalized Characteristic Path Length, also known as Small-Worldness, was calculated. This index is associated with both processing high efficiency and low wiring cost [35]. All global metrics describe brain abilities to process information in dedicated regions (segregation) and to coordinate this distributed processing to perform complex activities (integration).

With respect to local metrics, we considered Betweenness centrality, Nodal degree, and Nodal efficiency. Betweenness centrality is defined as the fraction of all the shortest paths passing through a given node; Nodal degree expresses the number of connections for each node; Nodal efficiency is inversely related to each node's paths length and identifies the less efficient nodes along certain routes. Higher values of these topological metrics identify key-nodes for brain integration and resilience. As explained above, we looked at a range of densities for each connectivity matrix and therefore obtained a range for each topological parameter, each corresponding to a different density value. First, we examined for each density value the differences between the two groups. Then, in order to obtain scalar values for each metric, the area under the curve (AUC) of the density distribution was calculated. For each considered global and local measure of connectivity, a two-sample *t*-test was used to assess group differences between DM1 patients and controls. For further analysis, as an alternative to the AUC approach, we averaged local and global measures across densities, to compute the “individual mean values” (Imv-). These global measures of connectivity (Imv-Clustering Coefficient (Imv-C), the Imv-Normalized Clustering Coefficient (Imv-NC), the Imv-Characteristic Path Length (Imv-PL), the Imv-Normalized Characteristic Path Length (Imv-NPL), the Imv-Small-Worldness (Imv-SWN), the Imv-Modularity (Imv-M) and the Imv-Assortativity (Imv-A), the Imv-Nodal degree (Imv-ND), the Imv-Betweenness centrality (Imv-BC), and the Imv-Nodal efficiency (Imv-NE)) were used for correlations with patients' MIRS/CTG triplets expansion (Bonferroni's correction: *α* = 0.05/2, *p* = 0.025) and, finally, with neuropsychological variables (Bonferroni's correction: *α* = 0.05/13, *p* = 0.004). Moreover, to define an appropriate statistical analysis, the Shapiro-Wilk test was used again.

## 3. Results

### 3.1. Demographic, Clinical, and Neuropsychological Characteristics

Patients and controls were not significantly different in age, gender, or years of formal education (*F*_1,55_ = 3.03, chi-square = 2.75, and* F*_1,55_ = 3.52, resp., all *p* = n.s.) (Table 1). Table 2 shows the genetic and clinical characteristics of all recruited patients. As reported in Table 2, most DM1 patients (19 out of 31, 61.2%) had an adulthood-onset of disease; 12 out of 31 patients (38.7%) had a childhood-onset. Following the guidelines of the Myotonic Dystrophy Consortium [22], one out of 31 patients (3.0%) was classified as E1 type, 15 out of 31 (48.4%) were classified as E2 type, 12 out of 31 (38.7%) were classified as E3 type, and finally 3 out of 31 (9.7%) were classified as E4 type. According to MIRS disease classification, 3 out of 31 (8.6%) were at the first stage of disease, 13 out of 31 (41.9%) were at the second disease stage, 11 out of 31 (35.4%) were at the third stage, and 4 out of 31 (12.9%) were at the fourth stage. CTG triplet expansion was negatively associated with years of formal education (*r* = −0.62, *p* = 0.002) and age at disease onset (*r* = −0.72, *p* < 0.001). No significant correlation was found between patients' MIRS and MMSE scores.

All patients with DM1 reported a normal MMSE score (range 26–30), though significantly lower than HS. Exploring specific cognitive domains (Table 3), DM1 patients performed significantly worse than controls on a visuospatial task only (Raven's Coloured Progressive Matrices;* F*_1,55_ = 16.1, *p* < 0.001).

### 3.2. Resting-State fMRI

#### 3.2.1. Network-Based Analysis

**Figure 1:**
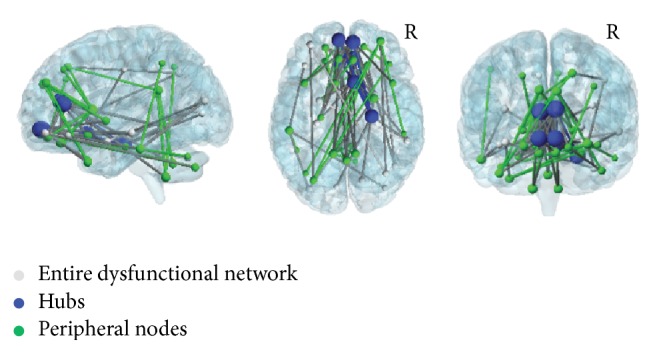
Reduced connectivity of DM1 brains obtained by network-based analysis. Widespread pattern of functional brain disconnection in patients with DM1 compared to healthy subjects (grey, blue, and green nodes). When Bonferroni's correction was applied to identify the most critical nodes of this dysfunctional network, two populations were identified, the “hubs” (blue) and the peripheral nodes (green). “Hubs,” characterized by the largest number of connections, were located in the anterior cingulum and in the orbitofrontal cortex bilaterally and in the right parahippocampal gyrus. Peripheral nodes (green), characterized by a smaller number of connections, were mainly located in the prefrontal, temporal, and parietal cortices and in the cerebellum. In the picture, node's size is proportional to the number of its connections. The brain network was visualized using the BrainNet Viewer (https://www.nitrc.org/projects/bnv/) [21]. See text for further details. R = Right.

NBS analysis showed a significant reduction of connectivity in patients with DM1 compared to HS in a large brain network formed by 51 different nodes and 83 edges (Figure 1, grey, blue, and green nodes together). Conversely, no significant reduction in connectivity was found in HS compared to DM1 patients. To isolate a more restricted pattern of disconnection (i.e., core of the dysfunctional network), we applied an extra Bonferroni's correction for multiple comparisons [*α* = 0.05/number of participants (*N* = 57), *p* = 0.0008]. This *p* value corresponds, in Student's *T* distribution table, to *t*-values ≥ 3.24, with 55 degrees of freedom (i.e, dof = *N* − 2, where *N* is the number of participants). The core of the dysfunctional network included therefore pairs of nodes whose statistical threshold corresponded to *t*-values ≥ 3.24, resulting in 37 nodes and 47 edges (Figure 1, blue and green nodes, and Table 4). Within this “core” dysfunctional network, the mean number of connections was 3.8 with a standard deviation of 2.5. According to Materials and Methods, we defined as “hubs” all nodes with at least 7 inter-Nodal connections.

Upon comparing DM1 patients to controls, dysfunctional “hubs” were located in the bilateral anterior cingulum (showing connections with 19 other brain regions), the orbitofrontal cortex (showing connections with 14 other brain regions), and the right parahippocampal gyrus (showing connections with 7 other regions). Those nodes, surviving Bonferroni's correction but whose number of inter-Nodal connectivity was lower than 7, were defined as peripheral nodes (Figure 1, green nodes). In the comparison between DM1 patients and controls, the peripheral nodes showing a significant reduction of connectivity included the prefrontal, temporal, parietal, and cerebellar regions.

In both groups, patients and controls, the normality of distribution of the extent of network's connectivity measure was not satisfied (in DM1: *W* = 0.75, *p* < 0.001; in HS: *W* = 0.63, *p* < 0.001). With respect to DM1 patients, for whom correlations were investigated between connectivity and cognitive performance, this extent of network's connectivity was positively associated with patients' scores at Raven's Coloured Progressive Matrices (*R* = 0.61, *p* = 0.001).

#### 3.2.2. Graph Theory Analysis

*Global Measures of Connectivity*. DM1 patients and controls did not differ in any considered AUC global measure. In both groups, patients and controls, the normality of distribution of the global connectivity measures was not satisfied (data not shown). In the DM1 group, we found significant positive correlations between MIRS scores and Imv-NC (*R* = 0.48; *p* = 0.014) and Imv-SWN (*R* = 0.52; *p* = 0.008) and a negative correlation between MIRS scores and the Imv-A (*R* = −0.46; *p* = 0.019).

**Figure 2:**
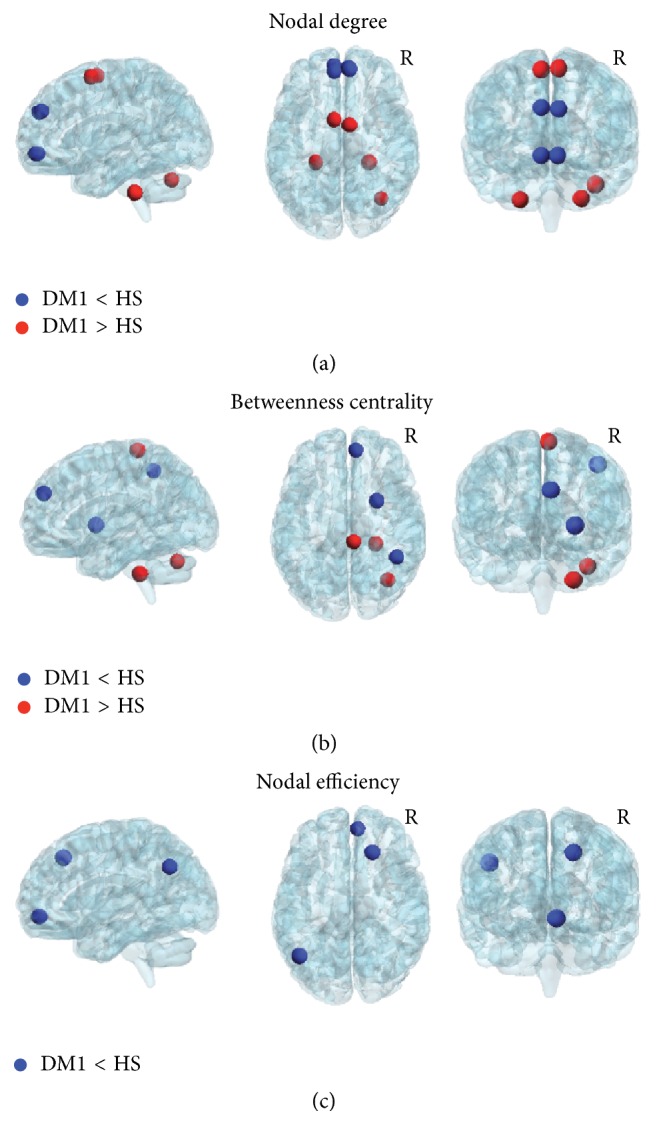
Abnormal topological properties of DM1 brains obtained by the graph theory analysis. Patients with DM1 compared to healthy subjects showed both decreased (blue nodes) and increased (red nodes) functional connectivity in centrality measures. Upon considering the Nodal degree (a) and Betweenness centrality (b) DM1 patients showed two distinct patterns, one located more anteriorly characterized by decreased connectivity (blue nodes) and one more posterior characterized by increased connectivity (red nodes). The former mainly involved the superior frontal and orbitofrontal gyri bilaterally. The latter involved the supplementary motor area and the cerebellum. Nodal efficiency (c) was reduced in the right superior frontal gyrus, in the right orbitofrontal gyrus and in the left angular gyrus. The brain network was visualized using the BrainNet Viewer (https://www.nitrc.org/projects/bnv/) [21]. See text for further details. R = Right.

*Local Measures of Connectivity*. Upon considering the Nodal degree (Figure 2(a), Supplementary Table, in Supplementary Material available online at http://dx.doi.org/10.1155/2016/2696085), DM1 patients showed a significant reduction in the superior frontal and in the orbitofrontal gyrus bilaterally. Conversely, this same measure was increased in DM1 patients compared to controls in the supplementary motor area, bilaterally, and in the cerebellum.

Upon considering Betweenness centrality (Figure 2(b), Supplementary Table), DM1 patients revealed reduced connectivity compared to controls in the right superior frontal gyrus, in the right inferior parietal gyrus, and in the right putamen. In contrast, DM1 patients showed a significant increase of connectivity in the right paracentral lobule, in the right CRUS-I, and in the right Lobule 10 of the cerebellum.

Moreover, upon considering Nodal efficiency (Figure 2(c), Supplementary Table), DM1 patients revealed reduced connectivity compared to controls in the right superior frontal gyrus, in the right orbitofrontal cortex, and in the left angular gyrus.

Finally, we found a significant negative correlation between the Nodal degree (Imv-ND) of right CRUS-1 and the patients' CTG triplet expansions (*R* = −0.54, *p* = 0.003).

## 4. Discussion 

Here, we provide the first evidence for widespread abnormalities in functional brain topological properties of DM1 brains. This fits with previous evidence of widespread structural alterations in DM1 patients, which are dominated by white matter involvement [7–11].

The majority of our patients had an adult or childhood disease onset, and their muscular impairments' severity was mild to moderate. Consistent with previous studies [6, 7, 10], there was no significant association between patients' CTG triplet expansion and MIRS scores, while a negative association was found between CTG triplet expansion and patients' age at disease onset. This indicates that a more severe genetic load associates with an earlier clinical onset, as supported by a previous study by our own group [7]. On the other hand, it is known that, in other neurological conditions, brain function does not linearly respond to the accumulation of structural abnormalities, with a significant modulation determined by environmental factors. In this view, topological brain characteristics (i.e., measures of networks' integrity and efficiency) might reflect better than structural information the global modifications occurring to DM1 brains.

We first performed NBS analysis, which revealed reduced connectivity in DM1 patients within a widespread brain network involving several nodes and edges, with the anterior cingulate and the orbitofrontal cortices as core regions. These regions are wired with several other nodes of the association cortex, thus playing the role of “hubs.” It is interesting to observe that although our DM1 patients performed within the range of normality in most cognitive tests, they reported poor scores at Raven's Coloured Progressive Matrices. This test assesses visuospatial reasoning, which is also regarded as a component of general intelligence [39], and mainly engages the orbitofrontal and parietal cortices [40]. Consistently, our DM1 patients' performance at Raven's Coloured Progressive Matrices test correlated with their extent of brain connectivity. Taken altogether, these results indicate that abnormal brain connectivity explains, at least partially, the patients' deficits in reasoning.

Upon considering graph theoretical measures, DM1 patients did not reveal significant differences in global topological indices with respect to controls. Changes to global measures typically reflect the presence of considerable brain disorganization such as that observed in patients with severe dementia [41]. The lack of significant reductions in global segregation/integration measures in DM1 patients [41] is therefore consistent with their relative preservation of general cognitive efficiency. Nevertheless, some global measures of connectivity (i.e., Assortativity, Normalized Clustering Coefficient, and Small-Worldness) were associated with patients' MIRS scores. The MIRS score returns a global measure of disability in DM1 patients. It is based on the assessment of progression of muscular impairment and, in principle, it should reflect also some contribution of CNS involvement. Indeed, it was previously shown that MIRS scores correlate with the extension of white matter damage in DM1 patients [7, 10]. Among global measures of connectivity, Assortativity [19], which was negatively associated with our DM1 patients' MIRS scores, has been previously regarded either as a measure of network vulnerability to pathological insults [42] or as an index of brain tissue resilience. Additionally, Normalized Clustering Coefficient and Small-Worldness, which were positively associated with our patients' MIRS scores, are considered as measures of network segregation and integration, respectively. Taken altogether, higher network segregation in the absence of a sufficient integration might reflect the presence of dysfunctional communication between nodes [20]. Considering this strict association between measures of global brain connectivity and patients' MIRS scores, we believe they might be useful biomarkers for patients' monitoring in clinical routine and trials.

Upon considering the local measures of brain connectivity, we found two distinct patterns in DM1 patients, namely, a more anterior decrease and a more posterior increase of connectivity. With respect to the anterior pattern, consistent with the findings obtained by NBS analysis, DM1 patients showed decreased connectivity in prefrontal (orbitofrontal and superior frontal gyrus) and parietal (inferior parietal and angular gyrus) regions. As mentioned above, these brain regions are implicated in higher-level functions and may account for patients' cognitive and behavioural symptoms [6, 7]. Consistently, a selective pattern of topological brain alterations, including Nodal degree, Betweenness centrality, and Nodal efficiency, was found in a selection of the nodes belonging to this network. This indicates that some of these nodes, which are the most “central” for an efficient transfer of information, are affected. The reduction of Nodal efficiency and Betweenness centrality that we found in patients' angular gyrus and inferior parietal gyrus, respectively, is only in apparent contrast with the increase of functional connectivity that we previously observed in a critical node of patients' default mode network [6]. These brain regions are located in neighbour but different brain areas, and these different findings seem to be complementary to each other rather than in contrast. Functional connectivity estimated by independent component analysis [6] returns a measure of segregation similarly to that expressed by Nodal degree and Clustering Coefficient as obtained by the graph theory. Conversely, Nodal efficiency and Betweenness centrality (found to be reduced here in DM1 patients) are regarded as measures of integration [43]. In this view, as previously suggested by van den Heuvel and Sporns [43], segregation and integration measures produce complementary information, and our previous and current findings are likely to detect different aspects of the pathophysiological processes occurring to DM1 brains.

With respect to the posterior pattern, DM1 patients showed increased connectivity in the supplementary motor area (SMA) and in the right cerebellum (CRUS-1 and lobule 10). Similar abnormalities have also been observed in patients with autism spectrum disorders (ASD) [44–46]. In this case, the atypical pattern of increased connectivity between the cerebellum and the sensorimotor areas was interpreted as a possible substrate for the repetitive stereotyped behaviours observed in autism [44]. Interestingly, autism-like traits have also been previously reported in patients with congenital DM1 [47]. Moreover, in the current work, we also found a significant association between patients' genetic load and their Nodal degree connectivity in CRUS-I. The SMA, the CRUS-1, and cerebellar lobule 10 are all regions implicated in “internally generated” planning of movements, in the planning of sequences of movements, and in motor coordination [48, 49]. We believe that the severity of abnormal connectivity within this set of regions might be relevant for the clinical interpretation of motor impairment in patients with DM1, which can differ across individuals. Motor impairment in DM1 is indeed predominantly due to muscular weakness. However, a recent fMRI study reported an association between myotonia and SMA activation in patients with DM1 [50], thus suggesting CNS contribution to patients' motor deficits [50]. In this view, we speculate that our findings of abnormally high connectivity within the motor network of DM1 patients might represent a compensatory, albeit nonefficient or maladaptive, mechanism of brain plasticity to contrast muscular impairment. This might be of clinical interest for tailored neurorehabilitation programs in DM1.

In conclusion, the current study describes, for the first time, the topological properties of brain functional networks in patients with DM1. Though preliminary, our data contribute to clarifying some relevant pathophysiological aspects of DM1, with a focus on both motor and nonmotor symptoms. Not only is this of speculative interest but it introduces a new potential tool for patients' assessment and monitoring. Finally, this work opens an important question on the possibility of implementing novel therapeutic approaches in clinical settings by using, for instance, the transcranial magnetic stimulation (TMS), or the transcranial direct current stimulation (TDCS). These techniques that exploit the unique plasticity of the human brain have already proven their usefulness in modulating brain connectivity and in improving the clinical outcome in various neurological patients [51–53].

## Supplementary Material

Mean value of local brain connectivity in DM1 patients and Controls.

## Figures and Tables

**Table 1:** Principal demographic characteristics of studied subjects.

	DM1 patients *N* = 31	HS *N* = 26	*p* value
Mean (SD) age [years]	39.9 (11.4)	45.7 (13.2)	n.s.^a^
Gender (F/M)	16.0/15.0	19.0/7.0	n.s.^b^
Mean (SD) years of formal education	12.4 (2.2)	14.0 (3.3)	n.s.^a^

^a^One-way ANOVA. ^b^Chi-square.

DM1 = myotonic dystrophy type 1; HS = healthy subjects.

**Table 2:** Principal genetic and clinical characteristics of DM1 patients.

	DM1 patients *N* = 31
*Age at onset:*	
Childhood-onset (age range: 6–16 years)	12 (38.7%)
Adulthood-onset (age range: 18–60 years)	19 (61.2%)

*Size of CTG triplets' expansion on DMPK gene:*	
Mean (SD) [range]	637.1 (456.4) [54–2000]

*IDMC nomenclature:*	
E1 (CTG range: 50–150) (*N* and %)	1 (3.0%)
E2 (CTG range: 151–500) (*N* and %)	15 (48.4%)
E3 (CTG range: 501–1000) (*N* and %)	12 (38.74%)
E4 (CTG range: > 1000) (*N* and %)	3 (9.7%)

*MIRS stage:*	
Stage 1 (*N* and %)	3 (9.7%)
Stage 2 (*N* and %)	13 (41.9%)
Stage 3 (*N* and %)	11 (35.5%)
Stage 4 (*N* and %)	4 (12.8%)

DM1 = myotonic dystrophy type 1; DMPK = myotonic dystrophy protein kinase; IDMC = international myotonic dystrophy consortium; and MIRS = muscular impairment rating scale.

**Table 3:** Performance obtained by DM1 and HS groups on neuropsychological testing.

*Domain *	DM1 patients	HS	*p* value^a^
Test/subtest	*N* = 31	*N* = 26	
*Cognitive efficiency*			
MMSE (normal cut-off ≥ 23.8)	27.9 (1.8)	29.7 (0.6)	0.000

*Verbal episodic long-term memory*			
15-word list:			
(i) Immediate recall (cut-off ≥ 28.5)	43.2 (6.0)	44.9 (9.5)	n.s.
(ii) Delayed recall (cut-off ≥ 4.6)	9.2 (3.2)	9.8 (2.8)	n.s.

*Verbal short-term memory*			
Digit span forward (cut-off ≥ 3.7)	5.3 (2.2)	6.1 (1.3)	n.s.
Digit span backward	4.9 (5.1)	5.1 (1.0)	n.s.

*Visuospatial short-term memory*			
Corsi span forward (cut-off ≥ 3.5)	5.3 (3.4)	5.5 (0.9)	n.s.
Corsi span backward	4.4 (1.5)	5.5 (0.9)	n.s.

*Language*			
Naming of objects (cut-off ≥ 22)	27.3 (6.4)	28.9 (1.3)	n.s.
Naming of verbs (cut-off ≥ 22)	25.4 (6.1)	26.1 (2.0)	n.s.

*Reasoning*			
Raven's Coloured Progressive Matrices (cut-off ≥ 18.9)	24.6 (8.6)	32.5 (2.6)	0.001

*Constructional praxis*			
Copy of drawings (cut-off ≥ 7.1)	9.9 (5.8)	11.3 (1.2)	n.s.
Copy of drawings with landmarks (cut-off ≥ 61.8)	57.4 (19.7)	69.0 (1.8)	n.s.

*Executive functions*			
Phonological word fluency (cut-off ≥ 17.3)	30.1 (17.7)	36.0 (8.4)	n.s.
Stroop-interference (cut-off ≤ 36.9)	30.2 (15.1)	—	—

DM1 = myotonic dystrophy type 1; HS = healthy subjects.

For each group of studied subjects, the table shows the performance scores means (SDs) obtained on neuropsychological testing. For each administered test, appropriate adjustments for gender, age, and education were applied according to the Italian normative data. Available cut-off scores of normality (≥95% of the lower tolerance limit of the normal population distribution) are also reported for each test. ^a^One-way ANOVA.

**Table 4:** Between-groups difference of functional connectivity into pairwise brain regions.

Pairwise brain regions	*t*-values^*∗*^
R superior frontal gyrus (orbital part) ↔ R middle frontal gyrus	3.67
L olfactory cortex ↔ L orbitofrontal gyrus	3.85
L orbitofrontal gyrus ↔ L rectus gyrus	4.70
L orbitofrontal gyrus ↔ R rectus gyrus	4.82
L superior frontal gyrus (orbital part) ↔ L cingulum (anterior part)	4.17
L olfactory cortex ↔ L cingulum (anterior part)	4.30
R olfactory cortex ↔ L cingulum (anterior part)	3.73
L rectus gyrus ↔ L cingulum (anterior part)	4.43
R rectus gyrus ↔ L cingulum (anterior part)	4.37
L superior frontal gyrus (orbital part) ↔ R cingulum (anterior part)	3.84
R superior frontal gyrus (orbital part) ↔ R cingulum (anterior part)	3.63
L olfactory cortex ↔ R cingulum (anterior part)	3.81
R rectus gyrus ↔ R cingulum (anterior part)	3.92
R orbitofrontal gyrus ↔ L parahippocampal gyrus	3.71
L orbitofrontal gyrus ↔ R parahippocampal gyrus	3.68
R orbitofrontal gyrus ↔ R parahippocampal gyrus	4.22
R olfactory cortex ↔ L amygdala	4.70
L orbitofrontal gyrus ↔ R amygdala	3.85
R orbitofrontal gyrus ↔ R amygdala	3.86
R amygdala ↔ L occipital gyrus	3.53
L superior frontal gyrus ↔ L caudate	3.68
R superior frontal gyrus ↔ L caudate	3.49
R rectus gyrus ↔ L caudate	3.72
L superior frontal gyrus (medial part) ↔ L pallidum	3.64
R orbitofrontal gyrus ↔ L pallidum	3.48
L superior frontal gyrus (medial part) ↔ R pallidum	3.61
R superior frontal gyrus (medial part) ↔ R pallidum	3.62
R parahippocampal gyrus ↔ R middle temporal gyrus	3.53
R amygdala ↔ R middle temporal gyrus	4.29
R superior frontal gyrus (medial part) ↔ L temporal pole	3.83
R orbitofrontal gyrus ↔ L temporal pole	4.44
L orbitofrontal gyrus ↔ L inferior temporal gyrus	3.60
L inferior parietal gyrus ↔ L inferior temporal gyrus	3.51
R orbitofrontal gyrus ↔ R inferior temporal gyrus	3.59
R orbitofrontal gyrus ↔ L cerebellum (Crus1)	3.84
R orbitofrontal gyrus ↔ L cerebellum (Crus2)	4.65
R inferior frontal gyrus ↔ L cerebellum (Crus2)	3.66
L cingulum (posterior part) ↔ L cerebellum (Lobule 9)	4.40
R cingulum (posterior part) ↔ L cerebellum (Lobule 9)	4.34
R precuneus R ↔ L cerebellum (Lobule 9)	3.55
L cingulum (anterior part) ↔ L cerebellum (Lobule 9)	4.12
R cingulum (anterior part) ↔ R cerebellum (Lobule 9)	3.66
L cingulum (posterior part) ↔ R cerebellum (Lobule 9)	4.32
R cingulum (posterior part) ↔ R cerebellum (Lobule 9)	3.53
L cingulum (anterior part) ↔ vermis	3.52
R cingulum (anterior part) ↔ vermis	3.47
L cingulum (posterior part) ↔ vermis	3.81
R cingulum (posterior part) ↔ vermis	3.58

^*∗*^Two-sample *t*-test with 55 degrees of freedom; *t*-values are reported. R = right; L = left; and ↔ = bidirectional connections.
