# Supplementary material for: Brain Connectomics' Modification to Clarify Motor and Nonmotor Features of Myotonic Dystrophy Type 1
Source: Neural Plast. 2016 May 25;2016:2696085. doi: 10.1155/2016/2696085 (PMC4897716; doi:10.1155/2016/2696085)
Supplement: Supplementary file 1 — Mean value of local brain connectivity in DM1 patients and Controls. [file 2696085.f1.docx]

**Supplementary Table. Mean values of local measures of brain functional connectivity in patients with DM1 and healthy subjects.**

| **Measures** | **Brain area** | **Side** | **DM1**  **(N=31)** | **HS**  **(N=26)** | **t-value** | **p-value*** |
| --- | --- | --- | --- | --- | --- | --- |
| **Nodal degree** | Superior Frontal gyrus (med.part) | L | 10.1 (1.9) | 11.9 (2.1) | -3.30 | 0.002 |
|  | Superior frontal gyrus (med. part) | R | 10.0 (1.9) | 12.2 (1.9) | -4.33 | 0.000 |
|  | Orbitofrontal gyrus (medial part) | L | 10.4 (2.1) | 11.8 (1.7) | -2.59 | 0.010 |
|  | Orbitofrontal gyrus (medial part) | R | 10.6 (1.9) | 12.2 (1.7) | -3.21 | 0.002 |
|  | Supplementary Motor area | L | 11.4 (2.1) | 10.0 (2.0) | 2.52 | 0.014 |
|  | Supplementary Motor area | R | 11.5 (1.7) | 10.3 (1.9) | 2.51 | 0.015 |
|  | CRUS-1 | R | 11.7 (1.9) | 10.1 (2.1) | 2.38 | 0.020 |
|  | Lobule 10 | L | 10.5 (2.2) | 9.0 (1.8) | 2.64 | 0.010 |
|  | Lobule 10 | R | 10.6 (1.8) | 9.4 (2.3) | 2.31 | 0.030 |
| **Betweenness centrality** | Superior frontal gyrus (med. part) | R | 19.8 (8.1) | 26.3 (11.9) | -2.45 | 0.017 |
|  | Inferior Parietal gyrus | R | 19.6 (9.3) | 25.1 (10.8) | -2.08 | 0.040 |
|  | Putamen | R | 22.6 (7.7) | 29.7 (13.0) | -2.54 | 0.013 |
|  | CRUS-1 | R | 28.6 (9.7) | 21.9 (10.0) | 2.55 | 0.013 |
|  | Lobule 10 | R | 25.0 (2.7) | 18.9 (8.7) | 2.07 | 0.042 |
|  | Paracentral lobule | R | 23.6 (1.2) | 15.0 (8.4) | 3.23 | 0.002 |
| **Nodal**  **efficiency** | Superior Frontal gyrus | R | 0.22 (0.1) | 0.23 (0.0) | -2.63 | 0.011 |
|  | Orbitofrontal gyrus (medial part) | R | 0.22 (0.0) | 0.23 (0.0) | -2.18 | 0.033 |
|  | Angular gyrus | L | 0.22 (0.1) | 0.23 (0.0) | -2-25 | 0.028 |

* Two-sample t-test with 55 degree of freedom

Mean, standard deviation (SD) t and p-values are reported. Abbreviations: DM1= Myotonic dystrophy type 1; HS= Healthy subjects. R=right; L=left.
